# Cystic Lymphangioma of the Mesentery in an Adult: A Case Report and Literature Review

**DOI:** 10.7759/cureus.63412

**Published:** 2024-06-28

**Authors:** Mohammed Mhand, Chafik Rhoul, Tariq Bouhout, Badr Serji

**Affiliations:** 1 Department of Surgical Oncology, Mohammed VI University Hospital, Regional Oncology Center, Faculty of Medicine and Pharmacy of Oujda, Oujda, MAR

**Keywords:** rare, case report, lymphangioma, abdominal mass, mesenteric cyst

## Abstract

Lymphangiomas are rare benign cystic tumors. Surgical excision is the primary treatment, aiming for complete removal. Diagnosis relies on imaging and histological confirmation. Malignant transformation is exceptionally rare. We report a 25-year-old man admitted for peri-umbilical abdominal pain and an abdominal mass. Imaging revealed multilocular peritoneal cystic formations with infiltration of adjacent mesenteric fat. Laboratory findings were unremarkable, and exploratory laparotomy was performed. A voluminous cystic mass originating from the mesentery was discovered, requiring intestinal sacrifice for complete resection. Immediate postoperative recovery was smooth. Pathological analysis confirmed the diagnosis of mesenteric cystic lymphangioma. The patient had a favorable outcome with no tumor recurrence at a three-year follow-up. We emphasize the significance of complete surgical removal to prevent complications associated with cystic lymphangioma and reduce the risk of recurrence.

## Introduction

Lymphangiomas are rare benign cystic tumors resulting from developmental abnormalities of the lymphatic system [1]. During embryogenesis, the failure of lymphatic channels to connect with the venous system results in the formation of an isolated lymphatic bud that eventually develops into a cyst [2]. Half of these lesions are congenital, and 90% of cystic lymphangiomas manifest by the age of two years [3]. Development in adults is exceptional [2,4].

The most commonly affected areas are the subcutaneous tissues of the neck, comprising about 75% of cases, and the armpits, comprising about 15% of cases [3]. Mediastinal and abdominal locations are much less frequent, representing roughly 10% of cases [3]. Cystic lymphangioma accounts for 7% of abdominal cystic lesions in adults [2,3]. Lesions mainly involve the mesentery but can also affect the gastrointestinal tract, spleen, liver, kidneys, adrenal glands, and pancreas [3].

When localized in the mesentery, the incidence is estimated at one in 100,000 in adults and one in 20,000 in children [5]. The sex ratio is one in adults, whereas, in children, it affects boys three times more frequently than girls [6,7].

Although often asymptomatic and incidentally discovered, the clinical manifestations of mesenteric lymphangioma are highly variable. These can include abdominal pain of varying locations, palpable mass, or abdominal distension. Sometimes, the clinical presentation can be acute (abdominal rigidity, obstruction) due to cyst complications (rupture, superinfection, intra-cystic hemorrhage, digestive volvulus) [8].

Diagnosis is suggested by imaging but requires histological confirmation. Malignant transformation is exceptional [9]. The treatment of choice is surgical, consisting of complete excision [5,10].

We followed the Surgical Case Report (SCARE) 2020 guidelines in the preparation of this report [11].

## Case presentation

This is a case report of a 25-year-old man with no notable medical history, who presented with peri-umbilical abdominal pain of a heavy nature for the past month, without other clinical signs. Clinical examination detected an abdominal mass, occupying the peri-umbilical region and right iliac fossa, evolving in the context of an afebrile state and preserved general condition.

Abdominal-pelvic ultrasound revealed the presence of an oval-shaped periumbilical peritoneal cyst and multiple well-defined cystic formations in the right iliac fossa.

**Figure 1:**
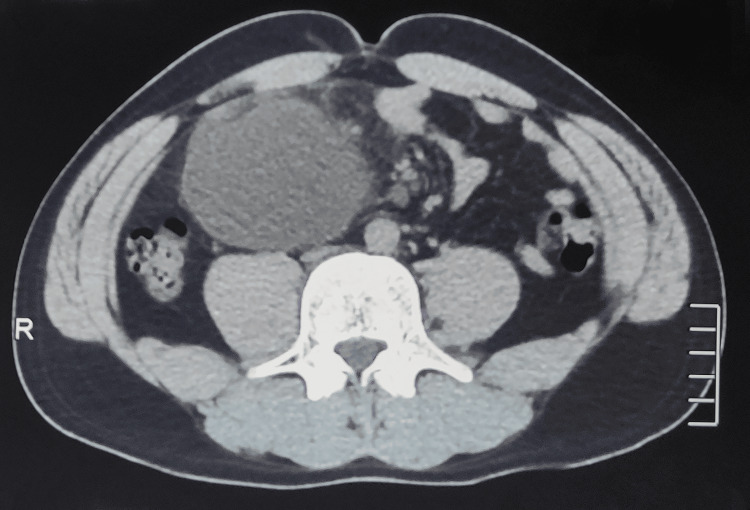
Abdominopelvic CT scan in the transverse section reveals a rounded cystic formation originating from the mesentery

An abdominal-pelvic CT scan was performed (Figure 1), revealing multilocular peritoneal cystic formations located in the right iliac fossa, with the largest measuring 90.5x39.5 mm. There was infiltration of the adjacent mesenteric fat, but no infiltration or involvement of nearby organs or blood vessels, and absence of deep lymph nodes or digestive thickening.

Laboratory findings showed no signs of anemia, inflammation, or infection. Specific diagnostic lab reports, including C-reactive protein (CRP), lactate dehydrogenase (LDH), and carcinoembryonic antigen (CEA) values, were all within normal ranges. Hydatid serology was negative for Echinococcus-specific antibodies, and exploratory laparotomy was decided.

**Figure 2:**
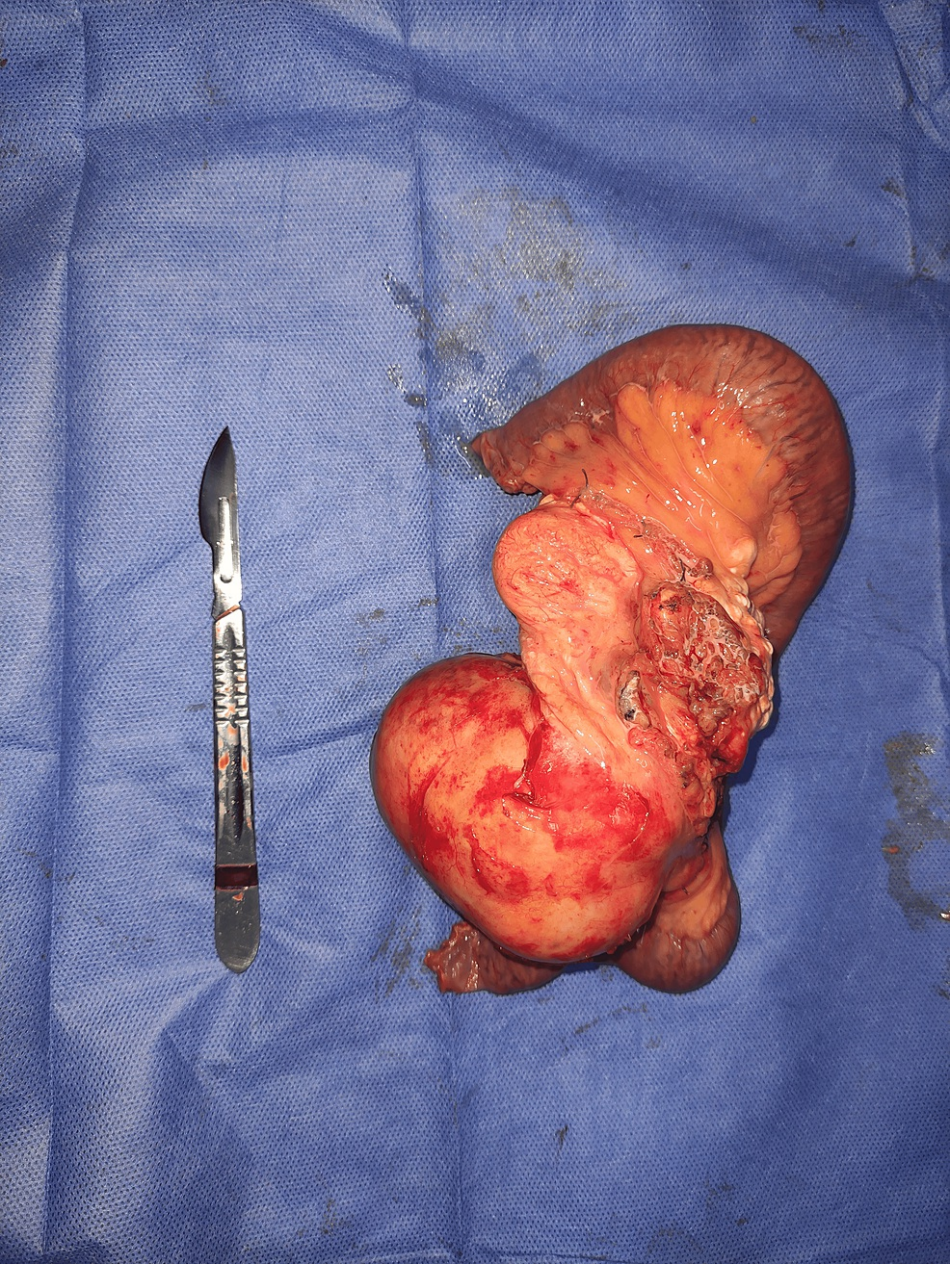
The surgical specimen of the resection, which includes the tumor

Exploration revealed a voluminous cystic mass originating from the mesentery (Figure 2), located 60 cm from the first ileal loop, extending 30 cm along the small intestine.

The appearance of the cystic mass was macroscopically benign with a thick wall. Complete resection could not be achieved without intestinal sacrifice. A monobloc resection was performed, removing both the mass and the adjacent intestinal segment, with manual ileo-ileal anastomosis.

**Figure 3:**
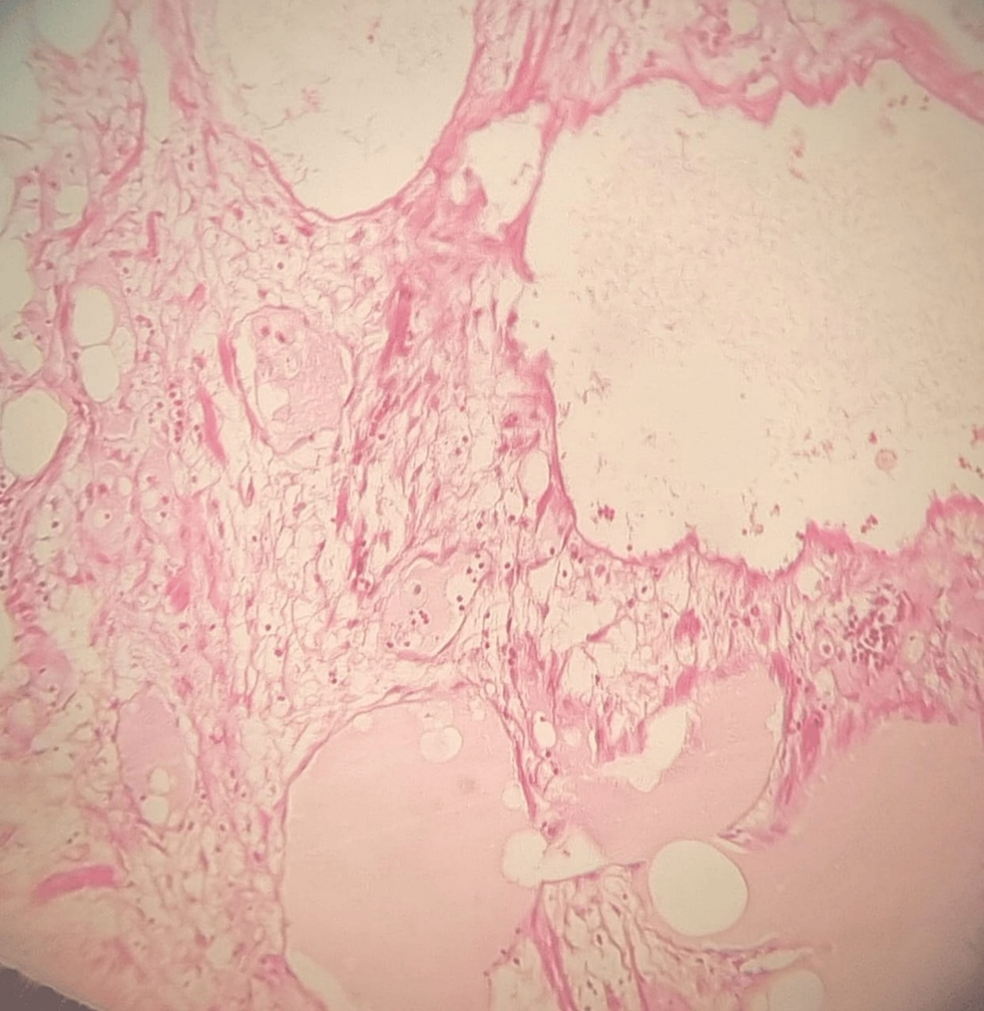
The microphotographic analysis showing dilated lymphatics filled with proteinaceous material; hematoxylin and eosin (H&E) stain x100

Immediate postoperative recovery was uneventful. Fluid aspirated from the cyst was serous and non-hemorrhagic. Histopathological examination of the operative specimen (Figure 3) revealed numerous dilated vascular cavities, lined by an outer connective tissue layer and an inner endothelial layer.

This endothelial lining confirms the vascular origin of the tumor, consistent with the diagnosis of mesenteric cystic lymphangioma devoid of malignant features. Post-operative management was uneventful, with no specific issues noted. The patient had a favorable outcome, with no tumor recurrence at a follow-up of three years.

## Discussion

Intra-abdominal mesenteric lymphangioma is an uncommon tumor typically diagnosed in early adulthood. The reported incidence of this tumor ranges from approximately one in 20,000 to one in 250,000 hospital admissions [12].

The tumor typically localizes in the subcutaneous tissues of the face and neck (60%), limbs (20%), thorax (10%), and axillary regions. Abdominal (2-10%) and thoracic (5%) localizations are less common [13].

Intra-abdominal lymphangiomas are predominantly located in the retroperitoneum, with the mesentery being the most common intraperitoneal site. Among intra-abdominal sites, the small bowel mesentery accounts for approximately 70% of cases, with approximately 50-60% of all cysts found in the ileal mesentery [14]. The etiology of mesenteric lymphangioma is congenital, resulting from abnormal embryonic development of the lymphatic system, which leads to the sequestration of lymphatic tissue. However, other potential causes have been suggested, such as abdominal trauma, lymphatic obstruction, inflammatory processes, surgery, and radiation therapy [1].

The clinical presentation of cystic lymphangiomas varies widely. Symptomatic lesions may manifest signs associated with tumor size or complications, varying from frequently asymptomatic masses in adults to acute abdominal pain or complications such as rupture, infection, intracystic hemorrhage, occlusion, torsion, compression, or infiltration of critical structures [15]. In terms of differential diagnosis, cystic lymphangiomas should be distinguished from other conditions, including ovarian cysts, digestive duplications, appendicular mucocele, and pancreatic cystadenoma [13].

Medical imaging has significantly advanced the diagnostic capabilities for this condition. Abdominal ultrasound, being non-irradiating and readily accessible, plays a crucial role and can aid in diagnosis even during the antenatal period [13]. An ultrasound examination typically reveals a cystic liquid lesion that is hypoechogenic, multilocular, with internal partitions, and characterized by a thick wall without vascularization on Doppler imaging [13].

Computed tomography (CT) imaging serves as the gold standard for diagnosing this condition, providing detailed information on tumor density, and its interaction with adjacent organs, and facilitating the distinction between retroperitoneal and intra-peritoneal lymphangiomas. Magnetic resonance imaging (MRI) is particularly useful for evaluating the cystic contents with high specificity facilitating precise diagnosis and offering detailed visualization of perivascular extension of the lesion [16]. Differential diagnosis on imaging may include cystic teratomas, hydatid cysts, or dermoid cysts [1].

Nevertheless, achieving a definitive diagnosis typically necessitates a pathological biopsy. This procedure reveals abnormally enlarged lymphatic vessels lined with flat endothelial cells, frequently accompanied by smooth muscle, blood vessels, fat, and lymphatic matrix [17].

Without intervention, the size of cystic lymphangiomas tends to increase over time, leading to compression of nearby tissues, blood vessels, nerves, and organs, ultimately causing associated complications [18].

In 1880, Tillaux conducted the initial successful removal of a mesenteric cystic tumor, and ever since, surgical resection has remained the primary treatment approach [19].

Efforts should be made to achieve complete excision while prioritizing organ preservation, given the benign nature of lymphangiomas [20]. If there is infiltration of the intestine or involvement of major mesenteric artery branches or adjacent organs, segmental intestinal resection may be necessary [21]. The prognosis following successful excision of mesenteric cystic tumors is generally excellent [1].

Recently, nonsurgical treatment methods have emerged as alternatives. Among these, sclerotherapy has become the most used approach for managing lymphangiomas that are challenging to completely resect or operate on. Compared to surgical resection, sclerotherapy offers the advantages of simplicity and reduced tissue injury. Additionally, several studies have demonstrated the effectiveness of sclerosing agents such as bleomycin sulfate, doxycycline, OK-432, acetic acid, and absolute alcohol in treating large cystic lymphangiomas, although their efficacy for microcystic lymphangiomas is more limited [18].

Nevertheless, sclerotherapy has been associated with certain adverse reactions, including airway obstruction and skin necrosis. Therefore, further evaluation of the efficacy and safety of sclerotherapy is warranted [18].

Another significant technique is radiofrequency ablation, which selectively targets and destroys diseased tissues using controlled heat, minimizing damage to surrounding tissues. Currently, radiofrequency ablation is considered a primary treatment option for microcystic lymphangiomas of the oral cavity and pharynx [18].

Targeted therapy, which selectively targets tumor cells while sparing normal cells, represents an innovative approach to the treatment of mesenteric cystic lymphangiomas. This method involves targeting specific lymphatic markers, such as Prox-1, VEGFR-3, PDGFR-β, and D2-40, especially in cases showing aggressive or recurrent behavior [20].

## Conclusions

Cystic lymphangioma is an uncommon lesion characterized by clinical and radiological variability. Its diagnosis can be challenging in adults, both before and during surgery. Differential diagnoses may include various cystic lesions such as simple mesenteric cysts, lymphatic malformations, hydatid cysts, and cystic teratomas. Imaging, particularly CT and MRI, plays a crucial role in delineating the extent of the lesion, assessing its interaction with adjacent structures, and guiding surgical planning. Complete surgical resection is regarded as the optimal treatment strategy for mesenteric lymphangiomas, with the definitive diagnosis confirmed through histopathology.
